# The anesthetic efficacy of ultrasound-guided lumbar plexus combined with quadratus lumborum block with Shamrock approach in total hip arthroplasty: study protocol for a randomized controlled trial

**DOI:** 10.1186/s13063-023-07619-z

**Published:** 2023-09-18

**Authors:** Xiaofeng Wang, Hui Zhang, Yongzhu Chen, Zhenwei Xie, Moxi Chen, Yonglin Chen, Junfeng Zhang

**Affiliations:** Department of Anesthesiology, Shanghai Jiao Tong University Affiliated Sixth People’s Hospital, No.600 Yishan Road, Xuhui District, Shanghai, 200233 China

**Keywords:** Analgesia, Hip arthroplasty, Lumbar plexus block, Quadratus lumborum block

## Abstract

**Introduction:**

The lumbar plexus originates from multiple segments of the spinal cord. Both single-level lumbar plexus block (LPB) and transmuscular quadratus lumborum block (TQLB) are commonly used to provide analgesia for the patients undergoing total hip arthroplasty (THA). However, neither of them can completely cover the lumbar plexus. Multiple-level LPB is also not recommended since this expert technique involves more potential risks. To achieve a better anesthetic effect and avoid risks, we propose to combine ultrasound-guided LPB with TQLB with Shamrock approach. We aim to assess the anesthetic efficacy of this combination technique and expect it will be an ideal alternative for conventional LPBs in THA.

**Methods and analysis:**

In this prospective randomized controlled trial, 84 patients schedule for THA will be enrolled. The patients will be randomly assigned at a 1:1:1 ratio to receive LPB at L3 level (P group), T12 paravertebral block combined with LPB at L3 and L4 levels (TP group), or LPB combined with TQLB at L3 level (PQ group). Each method will be evaluated in terms of the successful rate of sensory blockade, postoperative pain, performance time of block, requirement for intraoperative sufentanil, cumulative doses of intraoperative vasoactive medications, and adverse events.

**Ethics and dissemination:**

The study protocol has been approved by the institutional review board (IRB) at Shanghai Jiao Tong University Affiliated Sixth People’s Hospital, China (No.2020–031). The results will be disseminated in a peer-reviewed journal and the ClinicalTrials.gov registry.

**Trial registration:**

ClinicalTrials.gov, NCT04266236. Registered on 10 February 2020. ClinicalTrials.gov PRS: Record Summary NCT04266236.

**Supplementary Information:**

The online version contains supplementary material available at 10.1186/s13063-023-07619-z.

## Background

As a frequently used regional anesthesia technique for low limb surgery, lumbar plexus block (LPB) can provide effective analgesia and reduce opioid consumption for the patients undergoing total hip arthroplasty (THA) [1–3]. The lumbar plexus occasionally originates from T12 to L4. The three main branches of lumbar plexus that innervate the hip region, including the femoral, obturator, and lateral femoral cutaneous nerve, can be blocked with a single-level LPB at L3 or L4 [4, 5]. However, single-level LPB is difficult to completely cover the lumbar plexus. Strid et al. [6] observed the spread of local anesthetic with MRI in the volunteers underwent LPB at L4. The injectate was mainly confined between L2 and L4 and barely diffused to T12-L1. Thus, insufficient of analgesia of the incision area may occur due to the failure block of the branches derive from T12 and L1, such as iliohypogastric and subcostal nerve [7]. As we know, the effect of regional block depends on the coverage of related nerve branches at the surgical area. Therefore, to provide a more comprehensive coverage on the wide range of lumbar plexus, multiple-level block techniques, e.g., LPB at L2 and L3, at L3 and L4, or even combined with T12-L1 paravertebral block (PVB), were applied in some studies [8–11]. However, it is conceivable that these expert techniques may require more operator expertise, consume more performance time, increase the discomfort of the patients, and have a greater risk of complications [2, 12–14]. Thus, a both effective and convenient method should be investigated.

Transmuscular quadratus lumborum block (TQLB) could provide analgesia for the THA patients as well [15–17]. It might offer theoretical safety advantages over LPB due to the more superficial location of needle. Cadaveric studies supported when TQLB was performed, the dye injected between the quadratus lumborum and psoas major muscle would spread medially to the ventral rami of L1-L3 nerve roots. Meanwhile, the dye also spread laterally to iliohypogastric nerve (T12-L1) and subcostal nerve (T12) [18, 19]. However, other studies reported that the staining rate of obturator nerve and lateral femoral cutaneous nerve was merely 17% and 30%, respectively [20, 21]. In another RCT, the dermatomal coverage of TQLB was T8-L2 even with 40 mL injectate [22]. Sondekoppam et al. [23] also failed to block the femoral or obturator nerve distribution with TQLB at L3 in 4 patients, although they detected dye stain at L3 and L4 level in cadavers. These results indicate that cephalad levels of lumbar plexus (T12-L2) are more likely to be covered by TQLB and caudal levels (L3-L4) might be missed. Therefore, based on these observed outcomes, we consider to combine Shamrock approach LPB with TQLB at L3 level. This combination method has been proved effective in lower limb surgery in two pediatric case studies [24, 25]. However, its anesthetic efficacy in THA remains unproven.

Therefore, this RCT was designed to assess the anesthetic efficacy of ultrasound-guided LPB combined with TQLB with Shamrock approach for THA. We aim to identify whether this method can completely cover the lumbar plexus and achieve sufficient analgesia at the surgical area. We expect the outcomes will provide evidence for better clinical option of peripheral nerve block for THA.

## Methods

### Trial design, setting, and ethics

**Fig. 1:**
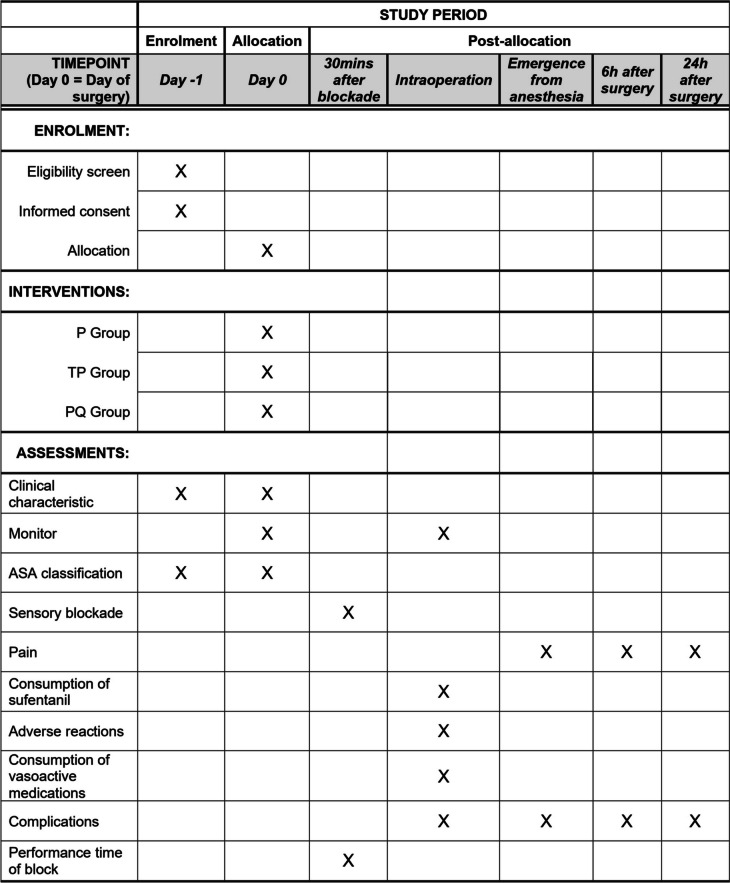
SPIRIT figure: schematic diagram of enrolment, interventions, and assessments

This prospective, randomized, superiority, observer-blinded controlled trial has been approved by the institutional review board (IRB) at Shanghai Jiao Tong University Affiliated Sixth People’s Hospital, China (No.2020–031), and registered prior to patient enrolment at ClinicalTrials.gov (NCT04266236, PI: XW, date of registration:10 February 2020). This protocol follows Standard Protocol Items: Recommendations for Interventional Trials (SPIRIT) 2013 Statements (Fig. 1 and Additional file 1).

### Participant recruitment and consent

The patients who present for elective unilateral THA will be screened in the preoperative interview. The potentially eligible patients will be contacted by the researcher (MC) who will explain the process of the study in detail to ensure that patients understand the entire clinical trial. In the meantime, they will receive the informed consents with detailed information including the purpose, procedures, contents of follow-up, data storage, benefits, and risks of the study and will be given adequate time to consider participation. Any question related to the study will be answered during the interview. The patients who willing to participant in the trial and sign the written informed consents will be recruited. They can withdraw at any point of time without depriving of any treatment and care. All the identifiable information will be confidential.

The inclusion criteria include the following:

- Patients aged between 18 and 75 years old without cognitive dysfunction
- Body mass index (BMI) between 18.5 and 30 kg/m^2^ and the weight  ≥ 50 kg
- American Society of Anesthesiologists (ASA) classification I-II
- Lateral operative incision approach

The exclusion criteria include the following:

- Refuse to general anesthesia (GA) with tracheal intubation
- Refuse to accept intravenous analgesic pump
- Nerve block is contraindicated due to various reasons, such as open trauma, hematoma or skin infection at the blocking area, lower limb neuromuscular disorders
- Coagulation dysfunction or anticoagulation therapy
- Known hypersensitivity or allergy to ropivacaine

### Randomization, allocation, and blinding

A simple randomization sequence was generated using the website http://www.randomization.com. The randomization list was stored in a password-protected file with restricted access only to the investigator (HZ) who prepared the list and would not be involved in the following research. Allocation details were sealed in sequentially numbered opaque envelopes (allocation ratio, 1:1:1). The envelope will be opened before surgery, and the participant will be assigned randomly to one of the three groups: (1) LPB at L3 level (P group); (2) T12 paravertebral block (PVB) combined with LPB at L3 and L4 levels (TP group); (3) LPB combined with TQLB at L3 level (PQ group) based on the allocation number. The entire intervention procedure including allocation will be finished in an isolated anesthetic room to keep the outcome assessor (YC) blinded. He is forbidden to ask any question about the intervention procedure. The anesthetists and surgeons in the operation will be kept blinded to the allocations as well. Emergency un-blinding rules will be applied if serious adverse event occur (e.g., cardiopulmonary arrest, cardiovascular collapse or local anesthetic systemic toxicity). Then, the patient will be withdrawn and the adverse event will be reported to the IRB.

### Patient and public involvement

Patients and the public were not involved in the design or planning of the study. Published results will be disseminated to the study participants on request.

### Interventions

**Fig. 2:**
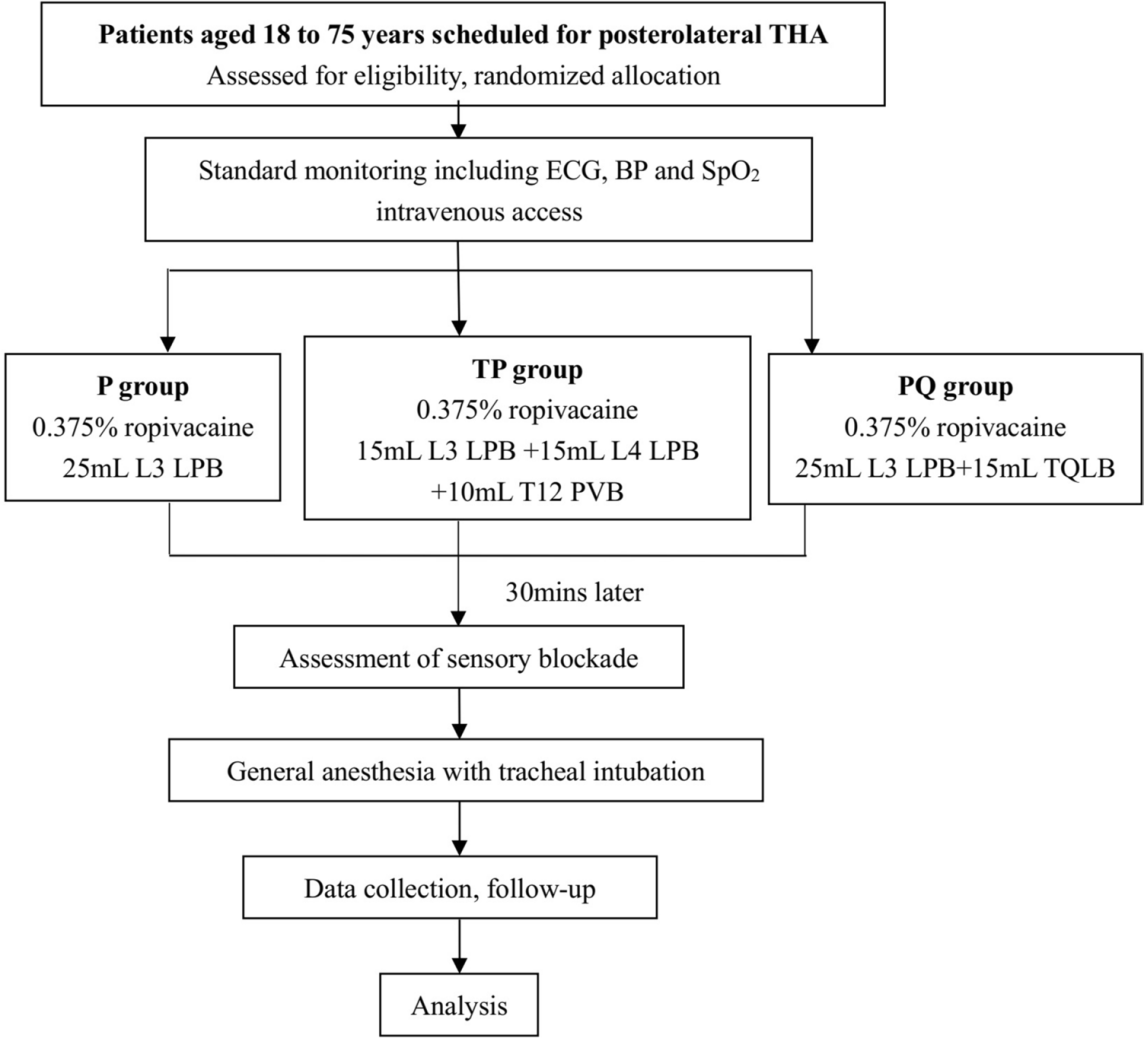
Flow diagram of trial procedures. THA, total hip arthroplasty; LPB, lumbar plexus block; PVB, paravertebral block; TQLB, transmuscular quadratus lumborum block

The trial flowchart is shown in Fig. 2. The intervention procedures will be performed by two unmasked operators (YC and ZX) with over 15 years of experience in ultrasound-guided regional anesthesia. They will be trained according to the following guidelines and strictly perform the nerve block procedures. The patients will be fasted for 8 h and water deprived for 2 h at least. Standard monitor will be applied to the patients who is transferred to the anesthetic room about 1 h before surgery, and the intravenous access will be established. No premedication will be given. The patients will be placed in the lateral decubitus position with both legs flexed. All the procedures will be performed using the in-plane technique with CX50 ultrasound system (Philips Healthcare Andover, MA, USA). A curve array probe (2–5 MHz) warped with a sterile cover and a 10-cm 22-gauge (G) block needle (KDL™, Kindly group, China) will be used. The spinous processes of T12 and L3-L5 should be determined by ultrasound scanning and palpation and then marked on the skin. After skin sterilization, 1 mL of 1% lidocaine for skin numbing will be given prior to each insertion. In addition, test injection with 1 mL of 0.9% normal saline will be given to confirm the correct localization of the needle tip and negative aspiration must be proved before each block.

In P group, LPB at L3 level will be administered with Shamrock approach, which is believed to be easier, faster to implement, and better to visualize the lumbar plexus compared with other LPB techniques [6, 26]. The probe will be placed transversely in the midaxillary line above the iliac crest and then shifted dorsally and tilted cephalad until the transverse process (TP) of L3 is identified. The quadratus lumborum muscle (QLM) is anterolateral to the apex of the TP with the psoas major (PM) anteriorly and the erector spinae muscle (ESM) posteriorly. When this Shamrock view obtained, the needle will be inserted in-plane and advanced in a posterolateral to anteromedial direction, targeting the hyperechoic lumbar plexus between the anterior and posterior lamina of the PM. Twenty-five milliliters of 0.375% ropivacaine (Raropin™, AstraZeneca AB, Sweden) will be injected.

In TP group, PVB at T12 combined with LPB at L3 and L4 will be performed as follows. Firstly, LPB at L3 and L4 (15 mL of 0.375% ropivacaine for both levels) will be performed with Shamrock approach as mentioned above. Thereafter, PVB at T12 will be conducted [27]. The probe will be placed vertically against T12 and then moved laterally. When the lateral border of the TP of T12, parietal pleura, 12th rib, and superior rib transverse process ligament are scanned, the probe should be moved slightly caudally to identify the thoracic paravertebral space (TPVS). The in-plane technique in a posterolateral to anteromedial direction will be used to inject 10 mL of 0.375% ropivacaine into the TPVS.

**Fig. 3:**
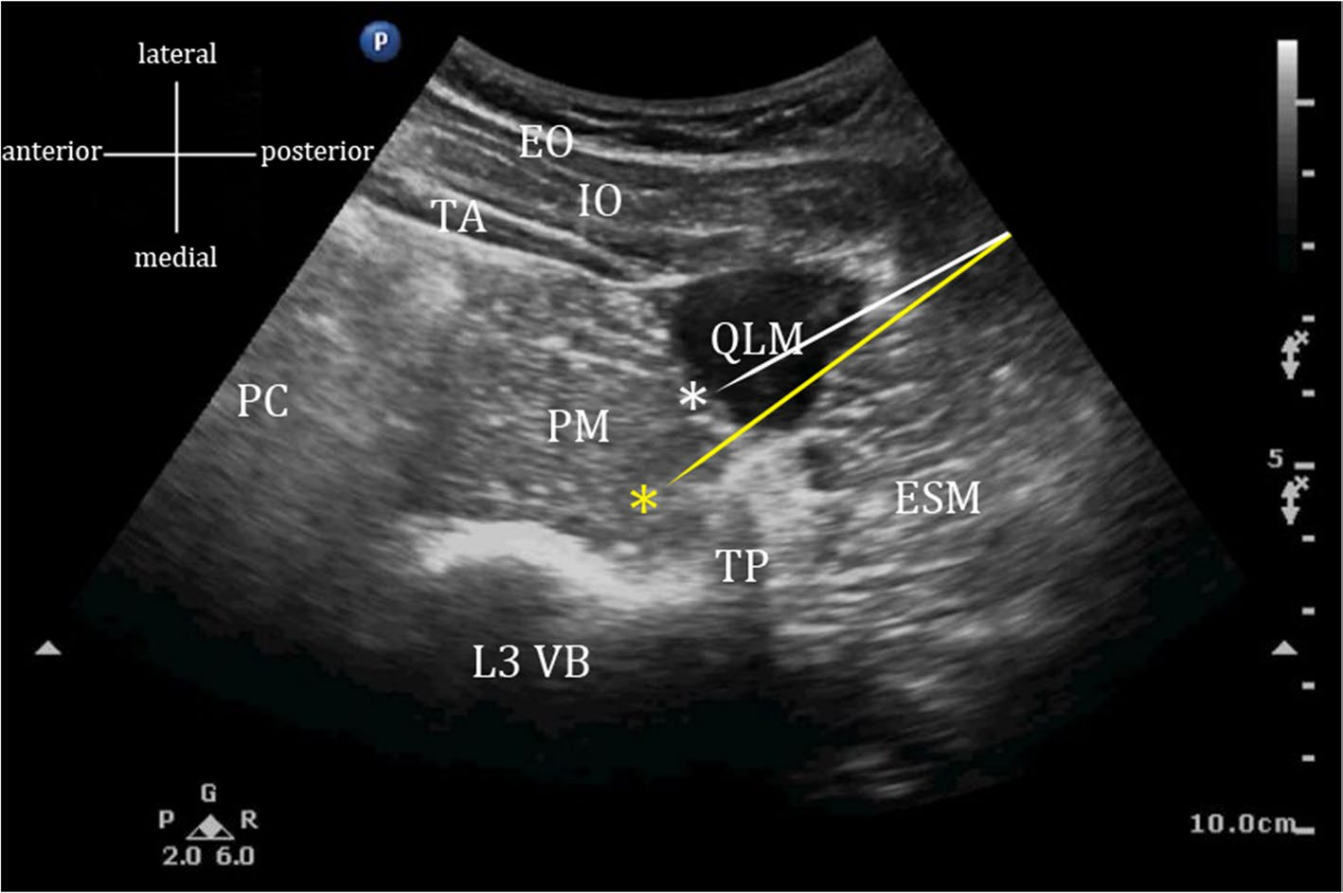
The ultrasound image of LPB combined with TQLB technique. White arrow and asterisk indicate needle trajectory and injection point of TQLB while yellow arrow and asterisk indicate those of LPB. ESM, erector spinae muscle; PM, psoas major muscles; QLM, quadratus lumborum muscle; L3 VB, L3 vertebral body; TP, transverse process; PC, peritoneal cavity. EO, external oblique; IO, internal oblique; TA, transversus abdominis

In PQ group, LPB combined with TQLB at L3 will be administered [25]. After LPB at L3 with Shamrock approach is performed as above (25 mL of 0.375% ropivacaine), the needle will be pulled back to the subcutaneous tissue, redirected and advanced through the QLM until the tip of the needle is located at the inter-fascial plane of the QLM and the PM (Fig. 3). Fifteen milliliters of 0.375% ropivacaine will be injected. Ideally, the QLM and PM should be separated as well as local anesthetic solution spreading along the anterior aspect of the QLM on the ultrasound image.

### Intraoperative anesthesia management and perioperative analgesia

The sensory at the surgical area will be assessed 30 min after blockade. Then, GA with tracheal intubation will be induced by intravenously administered propofol 2–3 mg/kg, sufentanil 0.5 μg/kg, and rocuronium 0.6 mg/kg. Inhaled sevoflurane (1–1.2 MAC) will be used to maintain anesthesia, and 5-μg increments of intravenous sufentanil will be given if required. ECG, BP, HR, RR, SpO_2_, and P_ET_CO_2_ will be recorded throughout the operation. Injection of intravenous ephedrine or deoxyepinephrine should be required when hypotension occurs (defined as a decrease of MAP more than 30% from baseline or SBP less than 90 mmHg). Atropine 0.5 mg will be injected intravenously when HR < 50 beats/min. After anesthesia emergence, the patient will be given a prophylactic dose of ondansetron 4 mg i.v to prevent nausea and vomiting and monitored in the post-anesthesia care unit (PACU) for at least 1 h. We will perform postoperative multimodal analgesia to each patient. Flurbiprofen 50 mg i.v will be given 10 min before the end of the surgery, and a Baxter elastomeric infusion pump containing 5 mg/mL tramadol with 0.16 mg/mL lornoxicam will be provided postoperatively at 2 mL/h (fixed dose, continuous infusion).

### Outcomes

#### Primary outcome evaluation

In order to evaluate the coverage range of local anesthetic, sensory blockades at 4 cutaneous areas, including lateral, anterior, and medial areas of thigh and lateral area of gluteus, will be scored according to a 3-point scale using a cold stimulation test, with relative comparison to sensation in the contra-lateral limb (0 = no block, 1 = analgesia, patient can feel touch but not cold, 2 = anesthesia, patient cannot feel cold or touch). The primary outcome will be the success rate of cutaneous sensory blockade. We define the sensory blockade as “success” with a score of 1 or 2 in every area and “inadequate” with a score of 0 in one area at least. Otherwise, the result will be defined as “failure” with a score of 0 in all areas.

#### Secondary outcomes evaluation

- Early postoperative pain scores including static pain and passive movement pain (at PACU, at 6 and 24 h after surgery) will be evaluated with visual analog scale (VAS) ranging from 0 to 10, where 0 means no pain and 10 means the worst pain
- Intraoperative consumption of sufentanil
- Intraoperative adverse reactions (hypotension, bradycardia, etc.)
- Intraoperative consumption of vasoactive medications (atropine, ephedrine and deoxyepinephrine, etc.)
- Complications related with anesthesia (local anesthetic systemic toxicity, pneumothorax, hematoma, etc.)
- Performance time of block (defined as the time from ultrasound scanning to the end of injection)

### Sample size calculation

Due to lack of referencedata in previous published study, calculation of the sample size was based on our pilot study with 24 subjects. We estimated the success rates of the three groups to be 20%, 70%, and 65%, respectively. Using chi-square test, sample size of 28 for each group will achieve 90% power to detect the difference with a two-tailed 5% significance level. The total sample size will be 84 including the possible dropouts.

### Data collection and management

All the data collected from electronic medical record, monitor machines, and relevant manual records will be recorded on the case report form (CRF) by the blinded research staff YC. Data entry, including quality checks and validation by double entry, will be performed with EpiData version 3.1. Missing unit record data will be compared with matching handwriting CRF and corrected accordingly. The data will be stored in a password-protected computer accessed only by YC.

### Statistical analysis

The analysis will be performed by intention-to-treat (ITT), and sensitivity analyses will include a per-protocol analysis. Since the study period is short (3 days), the loss rate to follow-up is expected to be low. Missing values will not be imputed and a complete-case framework will be used in that participants with missing values are listwise deleted. The statistical analysis will be performed with SPSS version 24 (IBM, Armonk, NY, USA). A two tailed, *P* value < 0.05 will be considered statistically significant. *P* value < 0.017 will be considered statistically significant after correcting for multiple comparisons. The demographic data will be expressed as mean ± SD (standard deviation), median and interquartile range (IQR, 25–75% percentile), or proportions (%) according to the type of variables. The Kolmogorov–Smirnov test will be used to assess the normality and homogeneity of the variables. Data for postoperative pain intensity, consumption of medications, and performance time, etc., will be analyzed by one-way ANOVA. Categorical variables, such as success rate and incidence of adverse reactions, will be analyzed by chi-square test or nonparametric test as appropriate. For multiple comparisons; Tukey in the ANOVA test; Bonferroni-corrected Mann Whitney *U* test in Kruskal Wallis; Bonferroni test will be used in chi-square test. In addition, analyze for the secondary outcome, early postoperative pain scores, will involve the use of linear mixed effects models because the participants will receive multiple observations.

### Harms

All the severe adverse events related to the study intervention will be recorded and reported as required to the IRB of Shanghai Jiao Tong University Affiliated Sixth People’s Hospital. Patients that are enrolled into the study will be covered by indemnity for negligent harm through the standard National Health Commission Indemnity arrangements.

### Auditing

No formal auditing process is proposed for this trial.

### Protocol amendments

Any change in this protocol must be agreed to by all the study investigators. Each amendment must be signed by the staff in the study and the amendment forms will be submitted to the IRB for approval. After that, the amendment will be updated in the clinical trial registry.

### Consent of assent

Written informed consents will be obtained by MC from all subjects before enrolment.

### Participant withdrawal and confidentiality

Participants may withdraw from the study at any stage and the reasons will be recorded. In addition, the private information of participants will be kept confidential to the public. The data collected will be stored securely for 5 years after the study finish.

### Dissemination policy

The outcomes of this feasibility trial will be disseminated in a peer-reviewed journal and the ClinicalTrials.gov registry. We will also be willing to share the results with our participants.

## Discussion

LPB is regarded as an ideal technique for postoperative pain control following THA. Despite this, which of the LPB techniques currently used is the best remains inconclusive. In this three-armed RCT, we propose to combine LPB with TQLB at L3 level with Shamrock approach. In theory, the local anesthetic injected at L3 level via LPB may spread in a longitudinal direction along the psoas muscle to block L2-L4 nerve roots. On the basis of LPB, TQLB may provide a better anesthetic effect for hip surgery via additionally blocking the branches derive from T12 and L1. The hypothesis will be verified through the results of our basic research questions: (1) What range of the lumbar plexus will be covered via this combination technique? (2) If complete cutaneous sensory block at the surgical area achieved, how about the analgesic effect, feasibility, and safety? Once the hypothesis is confirmed, associated benefits might be a better alternative to conventional LPBs, a reduced opioid consumption and a lower incidence of adverse reactions.

The previous studies mainly focused on evaluating the postoperative pain control of LPB or QLB or comparing between them. The pain score or analgesic consumption after the surgery was usually set as the primary outcome [16, 28, 29]. However, the analgesic effect indicates the results of multimodal analgesia and may be influenced by many factors. Our primary outcome concentrates on evaluating the success rate of block at the surgical area. It will help us identify the coverage of local anesthetic for lumbar plexus with the combination technique. On the other side, we will discuss the clinical effects in terms of opioid consumption, ease of operation, and safety through the secondary outcomes. The hemodynamic stability will be monitored considering the potential risk of extensive sympathetic blockade [30]. An effective, convenient, and safe technique is desirable, because many patients who undergo hip surgery are elderly with cardiovascular comorbidity. Thus, the eventual results can provide evidence for the superiority of combination method over conventional LPBs.

The limitation of the present study is that we have no direct evidence, such as MRI or anatomical support, to reveal what levels of lumbar plexus can be covered by the combination technique exactly. Instead, we can approximately understand the coverage of local anesthetic via the assessment of cutaneous sensory blockade because the 4 cutaneous areas related to the hip are innervated by different nerve branches [18, 31, 32]. Secondly, operator-related bias should be considered in the last analyze since blinding of operators is not possible. Lastly, some important outcomes, for instance, time to mobilization, postoperative opioid use, and length of stay are not included. They are not the main objectives of our study and will be investigated in further research.

To summarize, the results of this trial will help us to confirm the anesthetic efficacy of LPB combined with TQLB with Shamrock method in the patients undergoing THA. If our hypothesis is verified, this combination technique can be recommended as an effective and convenient alternative to the conventional LPBs.

## Trial status

At the time of manuscript submission, the study had been launched and a few patients had participated in the trial. The current version of protocol was 1.0 on March 25, 2020. The recruitment was begun on July 9, 2020, and anticipated to be completed in December 2023.

## Supplementary Information

**Additional file 1.** SPIRIT 2013 Checklist.

## Data Availability

The datasets used or analyzed during this study will be available from the corresponding author for reasonable purpose.
